# Immersive Virtual Reality to Restore Natural Long-Range Autocorrelations in Parkinson’s Disease Patients’ Gait During Treadmill Walking

**DOI:** 10.3389/fphys.2020.572063

**Published:** 2020-09-23

**Authors:** Alexis Lheureux, Julien Lebleu, Caroline Frisque, Corentin Sion, Gaëtan Stoquart, Thibault Warlop, Christine Detrembleur, Thierry Lejeune

**Affiliations:** ^1^Institute of NeuroScience, Université catholique de Louvain, Brussels, Belgium; ^2^Department of Physical and Rehabilitation Medicine, Cliniques universitaires Saint-Luc, Brussels, Belgium; ^3^Institut de Recherche Expérimentale et Clinique, Université catholique de Louvain, Brussels, Belgium

**Keywords:** Parkinson’s disease, gait disorders, gait assessment, virtual reality, gait variability, fractals, long range autocorrelations, treadmill walking

## Abstract

Effects of treadmill walking on Parkinson’s disease (PD) patients’ spatiotemporal gait parameters and stride duration variability, in terms of magnitude [coefficient of variation (CV)] and temporal organization [long range autocorrelations (LRA)], are known. Conversely, effects on PD gait of adding an optic flow during treadmill walking using a virtual reality headset, to get closer to an ecological walk, is unknown. This pilot study aimed to compare PD gait during three conditions: Overground Walking (OW), Treadmill Walking (TW), and immersive Virtual Reality on Treadmill Walking (iVRTW). Ten PD patients completed the three conditions at a comfortable speed. iVRTW consisted in walking at the same speed as TW while wearing a virtual reality headset reproducing an optic flow. Gait parameters assessed were: speed, step length, cadence, magnitude (CV) and temporal organization (evenly spaced averaged Detrended Fluctuation Analysis, α exponent) of stride duration variability. Motion sickness was assessed after TW and iVRTW using the Simulator Sickness Questionnaire (SSQ). Step length was greater (*p* = 0.008) and cadence lower (*p* = 0.009) during iVRTW compared to TW while CV was similar (*p* = 0.177). α exponent was similar during OW (0.77 ± 0.07) and iVRTW (0.76 ± 0.09) (*p* = 0.553). During TW, α exponent (0.85 ± 0.07) was higher than during OW (*p* = 0.039) and iVRTW (*p* = 0.016). SSQ was similar between TW and iVRTW (*p* = 0.809). iVRTW is tolerable, could optimize TW effects on spatiotemporal parameters while not increasing CV in PD. Furthermore, iVRTW could help to capture the natural LRA of PD gait in laboratory settings and could potentially be a challenging second step in PD gait rehabilitation.

## Introduction

Parkinson’s disease (PD) results from dopamine-producing neurons degeneration in the basal ganglia and is clinically characterized by classical motor triad combining rest tremor, plastic rigidity, and bradykinesia (Jankovic, 2008). Postural instability and gait disorders including reduced gait speed and step length, increased cadence and altered stride duration variability in terms of magnitude [increased coefficient of variation (CV)] and temporal organization constitute hallmarks of PD gait (Schaafsma et al., 2003; Warlop et al., 2016). Regarding temporal organization of gait, stride duration is known to fluctuate in a complex structured manner over many consecutive strides and this can be quantified with non-linear analysis such as long-range autocorrelations (LRA) computation (Hausdorff et al., 1995). Indeed, stride duration variability presents with a fractal pattern (Stergiou et al., 2006; Cavanaugh et al., 2017) that is somehow a sign of a long-term memory in the locomotor system (Hausdorff, 2007) and would be representative of adaptative abilities of healthy systems (Goldberger et al., 2002; Stergiou and Decker, 2011; Cavanaugh et al., 2017). On the contrary, a breakdown in LRA would be the signature of a pathological state (Goldberger et al., 2002; Stergiou and Decker, 2011; Ravi et al., 2020). Such a breakdown is present in PD (Ota et al., 2014; Warlop et al., 2016) with diminished fractal scaling exponent α that would be linked to the degeneration of the basal ganglia (Hausdorff et al., 1997; Goldberger et al., 2002; Hausdorff, 2007; Sarbaz et al., 2012) which are an important part of the central nervous system notably involved in the regulation of posture and gait (Takakusaki, 2017). Interestingly, strong correlations between breakdown of LRA and balance impairments but also with disease progression were recently highlighted in PD patients (Schaafsma et al., 2003; Ota et al., 2014; Warlop et al., 2016). The more the disease progresses, the greater the neurodegeneration, the lower the α exponent and the greater the risk of falls and their consequences. Therefore, LRA computation was proposed as an objective and quantitative biomarker of postural instability as well as disease progression that highly condition the higher fall risk inherently associated to PD (Hausdorff, 2009; Warlop et al., 2016).

Given the impact of these gait disorders on the risk of falling, researchers and clinicians are looking for innovative ways to assess and rehabilitate PD patients’ gait. Among others, treadmill walking is one of the most widely used tools to both assess and treat gait disorders in this population. Indeed, treadmill walking improves spatiotemporal gait parameters on the long term (Mehrholz et al., 2015) and is known to modify LRA in PD gait (Warlop et al., 2018; Hollman et al., 2020). In addition, this allows patients to walk long distances in a safe manner. However, treadmill walking lacks ecological visual inputs encountered when walking overground in daily-life situations. Indeed, combined with vestibular and proprioceptive information, visual inputs encountered during an ecological walk such as an optic flow (movement of the environment perceived while walking) are of essential importance to control gait. Previous studies stressed the unique importance of vision and especially optic flow during healthy subjects’ gait compared to the other sensory inputs concerning positional information (Patla et al., 2004; Chien et al., 2014) and correct adjustments of gait parameters during locomotion (Mukherjee et al., 2011). Likewise, visual disturbances lead to a higher variability of spatiotemporal parameters which may supposedly increase the risk of falling (Chien et al., 2014). If visual inputs are important for healthy subjects, it seems to be even more so for PD patients during walking (Azulay et al., 1999) and obstacle crossing (Vitório et al., 2013). This observation leads to think that it would be possible to further improve treadmill training by adding an optic flow. The most appropriate way to create a realistic optic flow during treadmill walking is to use high-end technologies such as immersive virtual reality (iVR) headsets.

In previous studies, 2D displays and projection systems were used to study the impact of optic flow during treadmill walking on healthy subjects’ gait (Prokop et al., 1997; Mohler et al., 2007; Katsavelis et al., 2010; Mukherjee et al., 2011; Chien et al., 2014, 2016) and PD gait (Schubert et al., 2005; van Wegen et al., 2006b). Although these devices are still used in recent studies, some of their technical characteristics do not make it possible to realistically produce an optic flow. Among others, these are: narrow vertical and horizontal field of view, no stereoscopic 3D rendering that gives the necessary depth of field essential to the optical flow, low rendering framerate limiting image fluidity, low immersion and feeling of presence in the virtual environment limiting the forgetting of the laboratory context in which the patient is located (Tieri et al., 2018). On the contrary, iVR headsets make it possible to reproduce this ecological visual information better than these older devices and seem to solve almost all their technical limitations.

Although only one previous study (Kim et al., 2017) showed both feasibility and tolerability of the iVR on treadmill in PD, no study has evaluated the impact on PD patients’ spatiotemporal gait parameters of an optic flow reproduced in an iVR headset during treadmill walking. Furthermore, no study has to date investigated the impact of this combination on gait variability measures (CV and LRA computation) of these patients, even with older devices. Thus, this pilot study aims to compare PD gait during three conditions: on overground and on a treadmill with and without iVR. Considering visual dependence of PD gait, the lack of optic flow on TW and recent technological advances, we put forward the hypothesis that the addition of an optic flow in iVR during treadmill walking will allow to more ecologically and accurately capture PD patients’ natural LRA in stride duration variability, similar to overground walking.

## Methods

This pilot study had approval from the local ethics committee (B403201837458/Clinicaltrial.gov registration: NCT04019158). Participants gave written informed consent in accordance with the Declaration of Helsinki prior to data collection. Testing took place in Cliniques universitaires Saint-Luc (Brussels, Belgium).

### Participants

Ten PD patients were recruited for this pilot study according to the following inclusion criteria: idiopathic PD according to the UK Brain Bank Criteria (Hughes et al., 1992), modified Hoehn & Yahr scale (Goetz et al., 2004) ≤ 3, Mini-Mental State Examination (Dick et al., 1984) > 24/30, no other pathology interacting with gait or causing dizziness, no uncorrected visual deficiency and ability to walk 512 consecutive strides (± 10–15 min). Patients were also evaluated using the Movement Disorders Society – Unified Parkinson’s Disease Rating Scale (Goetz et al., 2008), the Mini Balance Evaluation Systems Test (Franchignoni et al., 2010) and the Activities specific Balance Confidence scale (Powell and Myers, 1995).

Clinical characteristics and anthropometrics of the 10 PD patients are displayed in Table 1.

**TABLE 1:** Characteristics of the PD patients.

Women/men ratio	4/6
Age (y)	63.7(±10.6)
Modified H&Y scale (0–5)	1.75 [1–2]
MMSE (/30)	27.5(±1.8)
MDS-UPDRS total (/260)	44.7(±16.8)
MDS-UPDRS part III (/132)	24.9(±15)
Mini-BESTest (/28)	23.6(±1.9)
ABC-scale (%)	82.3(±14.6)

*Mean (± SD); Median [interquartile range]. ABC-scale: Activities specific Balance Confidence scale. H&Y: Hoehn & Yahr. MDS-UPDRS: Movement Disorder Society – Unified Parkinson’s Disease Rating Scale. Mini-BESTest: Mini Balance Evaluation Systems Test. MMSE: Mini Mentale State Examination.*

### Procedure

Participants were asked to walk in a randomized order in three conditions: Overground Walking (OW), Treadmill Walking (TW), and immersive Virtual Reality on Treadmill Walking (iVRTW). Each condition lasted 10–15 min in order to get a minimum of 512 strides necessary to determine the presence of LRA during gait with a high level of evidence (Crevecoeur et al., 2010; Warlop et al., 2017). During OW, patients walked on a 63.2 m rectangular track with smooth rounded corners at their comfortable speed. Prior to data collection, all patients performed one lap to get used to the terrain. In addition, patients were instructed to walk right in the middle of the track. During TW, patients walked on a treadmill at their comfortable speed assessed before with a 10 m walking test, were secured by a non-weight bearing harness and had no handrails that could have been grabbed so as not to interfere with the swinging of the arms. During iVRTW, patients walked on the treadmill at the same speed as during TW, wearing the harness, still without handrails, while wearing an iVR headset. Through the iVR headset (HTC, Vive, Taiwan), patients were immersed in a homemade environment created with Unity software (USA) and written in C# (Visual Studio, Microsoft, United States). The iVR headset weighed 470 grams and provided patients with a horizontal field of view of 110 degrees, a resolution of 1080 × 1200 pixels per eye and a refresh rate of 90 Hz. Also, this system allows to create depth of field by rendering stereoscopic 3D. To provide a complete visual immersion, the headset was designed to occlude peripheral vision in all directions. In this way, patients could not see the real environment around them and therefore only saw the virtual environment. The virtual environment consisted in a straight realistic endless hallway with some unevenly placed furniture on the sides in order to look real but to still avoid a rhythmic visual cueing to isolate optic flow effect. The goal was therefore to have a realistic ecological environment with fewer potentially distracting elements than an outside environment. Choosing a closed and restricted environment also saves computing power to ensure a fluid image without loss of framerate that could have given a jerky image. While walking on the treadmill, patients perceived an optic flow within the virtual environment moving at the same speed as the treadmill and creating the illusion of walking in an actual hallway (see Supplementary Material).

During each condition, two Inertial Measurement Units (IMeasureU Research, VICON, United Kingdom) were taped on patients’ lateral malleoli to record ankle accelerations at a sample of 500 Hz in antero-posterior direction. During OW, the recording was started after the warm-up lap. Regarding TW and iVRTW, recordings took place after a 3-min session of habituation to each condition and were initiated while patients walked on the treadmill while performing each condition. Data was then transferred onto a computer and stride durations were determined using a peak detection method (Terrier and Dériaz, 2011; Fortune et al., 2014).

Gait was assessed in terms of spatiotemporal gait parameters and variability in terms of magnitude and temporal organization.

Spatiotemporal gait parameters were assessed as follow:

- Gait speed (m.s^−1^) =
- $\text{During}\,\mathrm{OW}:\frac{\text{Total}\,\text{walking}\,\text{distance}\,\left(m\right)}{\text{Total}\,\text{acquisition}\,\text{time}\,\left(s\right)};$
- $\text{During}\text{TW}\text{and}\mathrm{iVRTW}:\left[cpsbreak\right]\mathrm{Present}\text{speed}\text{of}\text{the}\text{treadmill}\left(m.s^{-1}\right);$
$\text{Cadence}\left(\#\mathrm{steps}.\min^{-1}\right)=\frac{\text{Total}\,\text{number}\,\text{of}\,\text{steps}\,\left(\#\right)}{\text{Total}\,\text{acquisition}\,\text{time}\,\left(\min\right)};$
$\text{Step}\,\text{length}\,\left(m\right)=\frac{\text{Gait}\text{speed}\left(m.s^{-1}\right)}{\text{Cadence}\,\left(\mathrm{Hz}\right)};$
$\text{Mean}\,\text{stride}\,\text{duration}\,\left(s\right)=\frac{\text{Total}\,\text{acquisition}\,\text{time}\,\left(s\right)}{\text{Total}\,\text{number}\,\text{of}\,\text{strides}\,\left(\#\right)}$

To assess magnitude of stride duration variability, CV was calculated using the mean stride duration and SD:$CV\left(\%\right)=\left[\frac{S\,D}{m\,e\,a\,n}\right]\cdot 100$.

Temporal organization of stride duration variability was assessed by LRA computation using the evenly spaced averaged version of the detrended fluctuation analysis (DFA) (Almurad and Delignières, 2016) to obtain α exponent. This method was chosen given its robustness regarding stationary and non-stationary processes (Phinyomark et al., 2020; Ravi et al., 2020). LRA are present when α exponent values are between 0.5 and 1 meaning that large stride duration fluctuations tend to be followed by other large fluctuations, and vice-versa. An α exponent of 0.5 indicates the absence of LRA and a fully random organization (i.e., white noise). Also, α exponent values below 0.5 is the signature of anti-persistence. Finally, an α exponent of 1 (i.e., 1/f noise) is the boundary between stationarity and non-stationarity (Hausdorff et al., 1996). Peng et al. (1995) interpreted 1/f noise as “a “compromise” between the complete unpredictability of white noise (α = 0.5) (very rough “landscape”) and the very smooth “landscape” of Brownian noise (α = 1.5).” Then, 1/f noise is interpreted in the context of the theoretical framework of optimal movement variability (Harrison and Stergiou, 2015; Cavanaugh et al., 2017; Ravi et al., 2020) as the optimal state of variability characterizing healthy gait.

When using iVR, some studies reported the occurrence of what is known as “motion sickness.” This phenomenon is defined as the occurrence of adverse symptoms when using iVR headsets such as dizziness, nausea, headaches, and others (Cobb et al., 1999). Indeed, in some cases iVR can induce a mismatch between visual, proprioceptive, and vestibular inputs creating a sensory conflict (Reason, 1978). As such, patients of this study completed the Simulator Sickness Questionnaire (SSQ) after TW and iVRTW conditions to assess the presence of motion sickness based on a cut-off score of 15 out of 235.6 on the total score (Kennedy et al., 1993). Three sub-scores (i.e., Nausea, Oculomotor, and Disorientation) were also assessed (Kennedy et al., 1993).

### Statistical Analysis

A power analysis was computed using the data of Warlop et al. (2018). Authors compared PD patients’ temporal organization of stride duration variability using α exponent calculated with the DFA during two conditions: overground walking and treadmill walking. The power analysis was made using PASS software, in the idea of performing a one-way repeated measures ANOVA. Total sample of 10 participants achieved 80% power to detect differences among the means versus the alternative of equal means using an *F*-test with a 0.05 significance level. The size of the variation in the means was represented by their standard deviation which is 0.08. The common standard deviation within a condition was assumed to be 0.2.

Sigmaplot 13.0 software (Systat, Richmond, CA, United States) was used to analyze data. Normal distribution of the data was verified for all variables using the Shapiro–Wilk normality test. A paired *t*-test showed a difference between gait speed during OW and TW/iVRTW (*p* = 0.003). Given that gait speed influences spatiotemporal parameters and CV (Bollens et al., 2012; Warlop et al., 2016, 2018), OW could not be compared with TW and iVRTW. Since gait speed is not expected to modify the LRA (Bollens et al., 2012), a one way repeated measures ANOVA comparing the three conditions was performed only for the results of the evenly spaced averaged DFA and a Holm–Sidak *post hoc* test was performed. Regarding SSQ and the other gait outcomes, paired *t*-tests were performed to compare TW and iVRTW only. Cohen’s *d* was used to express the effect size between conditions for all outcomes if a significant difference was found.

## Results

**FIGURE 1:**
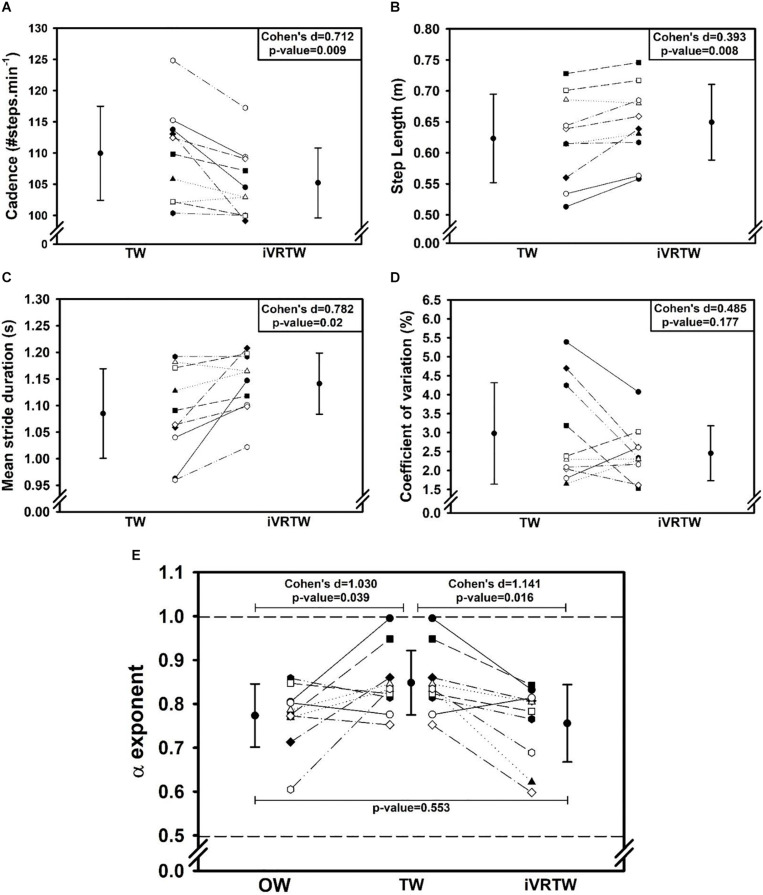
Graphs showing mean (±1 SD) and individual gait parameters values assessed during Overground Walking [OW, only graph (E)], Treadmill Walking (TW), and immersive Virtual Reality on Treadmill Walking (iVRTW) conditions. **(A)** Cadence graph. **(B)** Step length graph. **(C)** Mean stride duration graph. **(D)** Coefficient of variation graph. **(E)** α exponent graph (dashed lines corresponding to the limits between which there are LRA).

First, the added optic flow during iVRTW induced a positive effect on the spatiotemporal gait parameters with higher step length and reduced cadence compared to TW. Indeed, step length was higher (Cohen’s *d* = 0.392, *p* = 0.008) and cadence lower (Cohen’s *d* = 0.712, *p* = 0.009) during iVRTW than during TW for the same walking speed (Table 2 and Figure 1).

**TABLE 2:** Absolute mean values of the spatiotemporal gait parameters and stride duration variability assessed during Overground Walking (OW), Treadmill Walking (TW), and immersive Virtual Reality on Treadmill Walking (iVRTW) conditions and comparison between TW and iVRTW conditions.

	**OW**	**TW**	**iVRTW**	***p*-Value**
Speed (m.s^–1^)	1.31(0.10)	1.14(0.13)	1.14(0.13)	1.000
Cadence (#steps.min^–1^)	110.3(6.4)	109.9(7.5)	105.2(5.6)	**0.009**
Step length (m)	0.71(0.05)	0.62(0.07)	0.65(0.06)	**0.008**
Mean stride duration (s)	1.09(0.06)	1.08(0.08)	1.14(0.06)	**0.020**
Coefficient of variation (%)	1.95(0.53)	2.98(1.34)	2.46(0.72)	0.177
α exponent	0.77(0.07)	0.85(0.07)	0.76(0.09)	**0.016**

*Absolute value is expressed as mean (± SD). Bold data indicates if the comparison between TW and iVRTW (paired *t*-test) was significant (*p* < 0.05).*

Regarding magnitude of stride duration variability, the CV was similar between TW and iVRTW (*p* = 0.177) which shows that the added optic flow had no significant effect on this parameter (Table 3 and Figure 1).

**TABLE 3:** Absolute mean values of the Simulator Sickness Questionnaire (SSQ) total and sub scores for the comparison between Treadmill Walking (TW) and immersive Virtual Reality on Treadmill Walking (iVRTW) conditions.

	**TW**	**iVRTW**	***p*-Value**
SSQ total (/235.6)	15.0(19.7)	16.8(14.8)	0.809
SSQ nausea (/200.3)	11.5(12.6)	17.2(11.7)	0.370
SSQ oculomotor (/159.2)	12.1(15.7)	15.9(17.7)	0.563
SSQ disorientation (/292.3)	16.7(29.2)	8.4(9.7)	0.379

*Absolute value is expressed as mean (± SD).*

As expected, patients presented with a similar temporal organization of stride duration variability during OW and iVRTW. Conversely, α exponents were higher during TW. Indeed, even though all patients presented LRA during all conditions, α exponent was different between the three conditions [*F*(2,9): 5.633; *p* = 0.013]. While no difference was found between OW and iVRTW (*p* = 0.553), the α exponent during TW was higher than during OW (*p* = 0.039) and higher than during iVRTW (*p* = 0.016). The effect size was large for both comparisons: Cohen’s *d* = 1.030 when comparing TW to OW and 1.141 when comparing TW to iVRTW (Table 2 and Figure 1).

Regarding motion sickness, the SSQ total score as well as sub-scores were low and similar between TW and iVRTW (Table 3). Interestingly, 7/10 patients did not reach the cut-off score after TW and 5/10 after iVRTW while surprisingly no patient complained orally of motion sickness symptoms or discomfort linked to the headset during and after these two conditions. Note that two patients reached the cut-off score both after TW and after iVRTW, one patient who reached the cut-off after TW but not after iVRTW and three patients did not reach the cut-off after TW but did after iVRTW.

## Discussion

Purposes of this pilot study were to assess the effects of adding an optic flow displayed through an iVR headset during treadmill walking on gait and verify its feasibility and tolerability in PD population. The added optic flow during iVRTW improved step length and reduced gait cadence in comparison to TW at the same gait speed. Regarding magnitude of stride duration variability, CV was similar between iVRTW and TW. On the contrary, optic flow affected temporal organization of stride duration variability. Indeed, LRA were similar when the optic flow was present (during OW and iVRTW) but different when it was absent (during TW). Finally, this study confirmed that iVRTW is feasible and tolerable in PD.

Spatiotemporal gait parameters were influenced by iVRTW. Reduced step length is a well-known PD gait feature (Pistacchi et al., 2017), one of the components of cautions gait associated with fear of falling (Balash et al., 2007) and TW is known to be an effective rehabilitative approach to improve it (Mehrholz et al., 2015). However, it is also known that frail (Herman et al., 2005; Balash et al., 2007) and PD (Warlop et al., 2018) patients adopt a more precautious gait on the treadmill with decreased step length and increased cadence to maintain speed. This may have been due to the newness and the lack of “naturalness” of the task perceived by the patients. With the same gait speed as TW, patients increased step length and reduced cadence during iVRTW perhaps indicating a less cautious and a more natural gait adopted during this condition. The optic flow added with the iVR headset could then be an additional way to improve the well-known effect of TW on spatiotemporal parameters potentially leading to less cautious gait.

As described in the introduction, magnitude of stride duration variability (i.e., CV) is increased in PD (Schaafsma et al., 2003) and is associated with the presence of a postural reflex disorder (Ota et al., 2014). Also, usually considered as a marker of attentional load allocated to the task, CV was not different between TW and iVRTW. On one hand, some authors (Bello and Fernandez-Del-Olmo, 2012) hypothesized that the absence of optic flow during TW would decrease the attentional load on PD patients. With fewer environmental factors to consider while walking, PD patients would then more easily focus on walking on the treadmill allowing to overcome damaged automaticity in PD gait (Baker et al., 2007). If this hypothesis was accurate, CV would have been higher during iVRTW than during TW since iVRTW can be disturbing at first glance for patients (newness of the task, safety instructions to follow, potential anxiety of not seeing the real environment). On the other hand, recent studies showed that walking speed has the greatest influence on CV in both healthy and PD populations and, for the latter, for both OW and TW (Bollens et al., 2012; Warlop et al., 2018). Since CV was similar between TW and iVRTW for the same gait speed, it could be concluded that the magnitude of stride duration variability does not seem to be influenced by the presumed attentional load induced by the added optic flow in iVR. Note that such results are also in line with a similar study conducted on young healthy subjects using older equipment (Katsavelis et al., 2010).

In contrast to the magnitude of stride duration variability, the temporal organization of gait variability was significantly modulated by the addition of optic flow during iVRTW. Such results brought two lines of thought. On one hand, this study confirmed that TW would improve the temporal organization of gait variability among PD (Warlop et al., 2018; Hollman et al., 2020). On the other hand, by adding an optic flow, iVRTW would be a controlled and safe alternative to assess and maybe rehabilitate PD patients’ gait in a more ecological way.

All patients exhibited α exponents between 0.5 and 1 in all conditions, indicating the presence of LRA in the temporal organization of stride duration variability. Interestingly, α exponent during TW was closer to 1, approaching 1/f noise considered as an optimal state of variability (Peng et al., 1995; Harrison and Stergiou, 2015; Ravi et al., 2020) and indicating strong coordination among the multiple sub-systems that regulate locomotion (Stergiou and Decker, 2011; Delignieres and Marmelat, 2012). Attempting to get closer to 1/f noise seems clinically significant given the results of previous studies. Indeed, Hausdorff (2007) stated that it was possible to discriminate between elderly fallers (α close to 0.5) and non-fallers (α close to 1) based on LRA computation and that this could even have a prognostic value on the risk of falling. Similarly, a positive correlation was shown between low α exponent and poor balance test scores in PD patients (Ota et al., 2014; Warlop et al., 2016). So, getting closer to 1 seems to be an interesting goal in PD patients’ gait rehabilitation. On the other hand, α exponent was lower and similar between OW and iVRTW. Optic flow could be an explanatory factor since PD patients can overreact to visual information for maintaining balance (Schubert et al., 2005; Snijders et al., 2011). By increasing degrees of freedom, the added optic flow could thus increase the regulatory load on the locomotor system (Katsavelis et al., 2010) during iVRTW in a similar way to OW, decreasing α exponent compared to TW. On the contrary, the absence of optic flow during TW reduces the amount of information to be managed by the patients, allowing them to focus on the motor task. In addition to previous studies (Warlop et al., 2018; Hollman et al., 2020), this study highlights that, by reducing the degrees of freedom on the PD deficient locomotor system, absence of optic flow combined with the constant rolling of the treadmill could provide a framework for PD patients to regulate the temporal organization of their gait. The treadmill would provide external cues to patients allowing to bypass defective internal pallidocortical projections responsible for rhythm control impairments in PD (van Wegen et al., 2006a; Baker et al., 2007). It is therefore interesting to notice that during iVRTW, the rolling of the belt was kept as well as the constant speed imposed. Therefore, patients should somehow have kept this framework and this cueing effect, but these seemed to be lost by the addition of the optic flow. This may further underscore the fact that PD patients take into account and rely predominantly on visual information and optic flow to regulate their gait (Azulay et al., 1999; Vitório et al., 2013) compared to proprioceptive information, whose acuity is diminished in PD (Almeida et al., 2005; Konczak et al., 2009). From a PD gait rehabilitation point of view, TW could be conceived as a first step and iVRTW could have the potential to be a second stage in a challenging, safe and ecological rehabilitation approach, perhaps allowing a smoother progression between what has been worked on during rehabilitation and everyday walking.

Likewise, a previous study questioned the adequate gait assessment of gait variability during TW in PD participants (Warlop et al., 2018). As the study of LRA has to be done over a long period of time, much research has relied on treadmill walking in laboratory setting to assess PD patients’ temporal organization of gait in a controlled and safe manner. However, the results of the present study and of previous ones (Warlop et al., 2018; Hollman et al., 2020) highlight that the assessment of LRA on a treadmill does not adequately capture the natural temporal organization of gait in PD patients. Conversely, iVRTW could be an interesting way since α exponent during iVRTW was similar to that of OW.

Results regarding motion sickness are in line with those of the sole previous study conducted on PD population (Kim et al., 2017). Indeed, five patients out of 10 reached the cut-off point at which motion sickness is considered significant after iVRTW. However, this cut-off was already reached by three patients after TW. The fact that motion sickness symptoms appeared even without iVR for some PD patients was expected because it is well-known that these patients have impairments in sensory processing and integration (Hwang et al., 2016). These impairments coupled with medication (Chaudhuri and Schapira, 2009) could then be explanatory factors. Likewise, no significant differences were found regarding the SSQ total score and sub-scores between the two conditions meaning that iVRTW did not induce more motion sickness than TW. The hypothesis behind this result is that the mismatch between the visual, proprioceptive and vestibular inputs perceived by the patients was minor during iVRTW. Given that motion sickness is induced by a sensory conflict (Reason, 1978), it could be concluded that the iVR method of displaying an optic flow during treadmill walking used in this study would be efficient enough to limit this conflict. Another explanatory factor could be the technological upgrade seen regarding iVR in the last 10 years: better image framerate, higher display resolution, higher field of view. Furthermore, this present study reinforces the results of the sole previous study (Kim et al., 2017) who had shown tolerability over 5 min sessions while the present results showed tolerability over a ± 15 min session.

Some limitations of the present study should be addressed. First, although significant results were found only 10 PD patients were included. This study should be considered as a pilot study and it could be used to make sample calculations for future studies on the subject. Second, included patients were clinically mildly affected by PD with a fairly low Hoehn & Yahr score. More transversal and longitudinal studies including more patients at different stages of the disease are then required to confirm our findings regarding spatiotemporal gait parameters and on magnitude and temporal organization of stride duration variability. Third, one of the safety instructions during iVRTW was to follow a virtual security fence in iVR ensuring the patients walked at the right speed on the treadmill. This may have caused a cueing effect that may have had consequences on patients’ gait. Fourth, this study only investigated the direct adaptation of patients during TW and iVRTW but did not investigate potential after-effects when walking overground directly after these two conditions. Future studies should therefore focus on the acute effects potentially present after these particular walking conditions. Fifth, despite the impressive technological advancement of iVR headsets, the 2 years old headset used in this study only proposes a horizontal field of view of 110 degrees. Although it is superior to the devices used in previous studies, it is still below the natural field of view of the human eye. This loss of peripheral vision could have a significant impact on the correct perception of the optic flow during iVRTW. Newer headsets already offer a significant improvement in display resolution and a larger field of view. It is then likely that iVR headsets with a natural field of view will be available in a few years’ time. Finally, this study focused solely on a single method to analyze gait dynamics. Future studies similar to this one could add other methods to analyze gait dynamics (ARFIMA, Lyapunov exponent, entropy) (Roume et al., 2019; Ricaurte et al., 2020).

In conclusion, this pilot study highlighted that iVRTW could enhance the effectiveness of TW in improving step length while not increasing magnitude of stride duration variability. Also, TW could be perceived as a first step in PD gait rehabilitation to regulate temporal organization of gait and iVRTW as a second one within a challenging, safe and ecological gait rehabilitative approach. In addition, iVRTW may be a more adequate way to safely assess LRA in PD gait over a long period of time than TW. Future transversal and longitudinal studies including more PD patients presenting with a broader spectrum of disease severity need to be conducted in order to confirm these findings.

## Data Availability Statement

The raw data supporting the conclusions of this article will be made available by the authors, without undue reservation.

## Ethics Statement

The studies involving human participants were reviewed and approved by Comité d’Ethique Hospitalo-Facultaire of Cliniques universitaires Saint-Luc. The patients/participants provided their written informed consent to participate in this study.

## Author Contributions

AL handled the creation of the iVR environment and of the study protocol, managed patient recruitment, data collection and analysis, interpretation of the results, and wrote the manuscript. JL provided methodological inputs to the study, participated in the interpretation of the results, and revised the manuscript. CF and CS helped with patient recruitment, data collection, data analysis, and interpretation of the results. GS helped with patient recruitment, participated in the interpretation of the results, and revised the manuscript. TW participated in the interpretation of the results and revised the manuscript. CD provided methodological and statistical inputs to the study, helped to the application of mathematical methods, participated in the interpretation of the results, and revised the manuscript. TL provided methodological inputs to the study, participated in the interpretation of the results, greatly assisted in writing the manuscript, and revised the final manuscript. Lastly, all authors approved the final manuscript.

## Conflict of Interest

The authors declare that the research was conducted in the absence of any commercial or financial relationships that could be construed as a potential conflict of interest.
