# Supplementary material for: Immersive Virtual Reality to Restore Natural Long-Range Autocorrelations in Parkinson’s Disease Patients’ Gait During Treadmill Walking
Source: Front Physiol. 2020 Sep 23;11:572063. doi: 10.3389/fphys.2020.572063 (PMC7538859; doi:10.3389/fphys.2020.572063)
Supplement: Supplementary file 1 [file Table_1.DOCX]

| **(A)**   | |
| --- | --- |
| **(B)**  **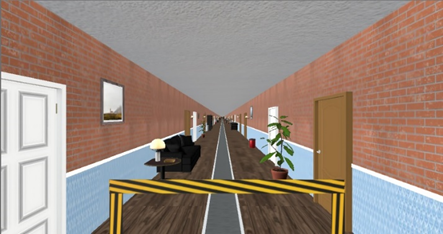** | **(C)**  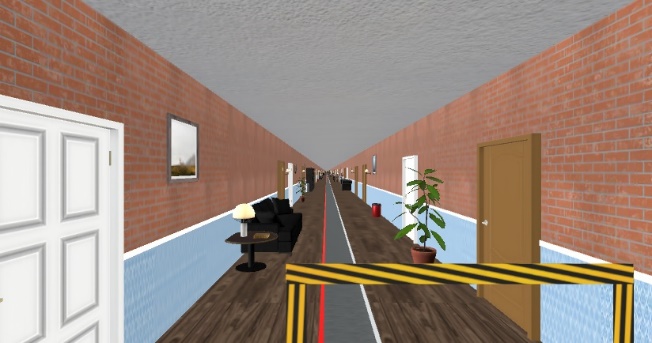 |
| **(D)**  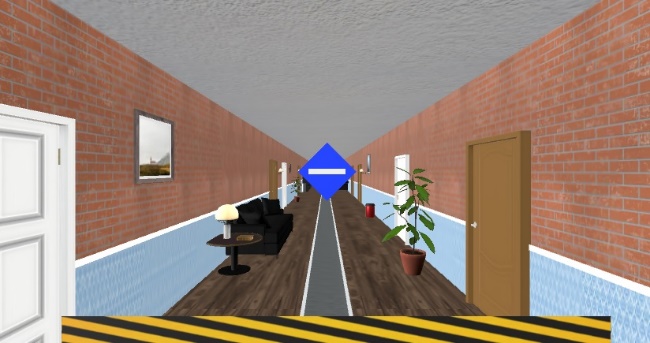 | **(E)**  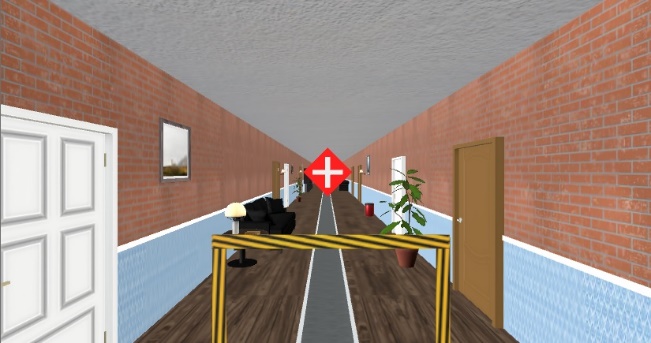 |

**Supplementary material 1.** iVRTW setting and safety rules. **(A)** Patient walking on the treadmill wearing a non-weight bearing security harness (Volt, Petzl, France) attached to the ceiling and an immersive virtual reality (iVR) headset (Vive, HTC, Taïwan) (1). The patient sees a long hallway moving forward at the same speed as the speed of the treadmill (2). **(B)** What the patient sees if the security rules are followed. To walk in the middle of the treadmill, the patient must walk on the grey carpet between the two white lines on the floor representing the left and right limits of the treadmill. **(C)** If the patient is walking too much on one side (on the left in the example), the line corresponding to this side turns red. The line turns back to white if the patient recenters himself in the middle. The yellow and black lined fence always represents the front end of the treadmill and is moving forward as fast as the optic flow. **(D)** If the patient is walking too fast on the treadmill and is too close to the fence, a blue panel with a “-“ sign appears and a voice urges the patient to walk slower. **(E)** If the patient is walking too slowly and is too far from the fence, a red panel with a “+” sign appears and a voice urges the patient to walk faster. The panels disappear if the patient walks back to the right speed. The width between the white lines and the width of the grey carpet, the height of the fence and the distance between the front and the back limits can be modulated according to any treadmill dimensions using an option interface. The speed of the optic flow and the fence can also be changed using this option interface.
